# Who is who in cardiovascular research? What a review of Nobel Prize nominations reveals about scientific trends

**DOI:** 10.1007/s00392-021-01813-2

**Published:** 2021-03-06

**Authors:** Marie Drobietz, Adrian Loerbroks, Nils Hansson

**Affiliations:** 1 Department for the History, Philosophy, and Ethics of Medicine, Faculty of Medicine, Heinrich-Heine-University, Moorenstr. 5, 40225 Duesseldorf, Germany; 2 Institute for Occupational, Social and Environmental Medicine, Faculty of Medicine, Heinrich-Heine-University, Moorenstr. 5, 40225 Duesseldorf, Germany

**Keywords:** Nobel Prize, Excellence in cardiovascular research, Helen B. Taussig, Cardiology, Cardiac surgery

## Abstract

**Background:**

Since 1901, at least 15 scholars who contributed to cardiovascular research have received a Nobel prize in physiology or medicine.

**Methods:**

Using the Nobel nomination database (nobelprize.org), which contains 5950 nominations in the accessible period from 1901 to 1953 in physiology or medicine, we listed all international nominees who contributed to cardiovascular research. We subsequently collected nomination letters and jury reports of the prime candidates from the archive of the Nobel Committee in Sweden to identify shortlisted candidates*.*

**Results:**

The five most frequently nominated researchers with cardiovascular connections from 1901 to 1953 were, in descending order, the surgeon René Leriche (1879–1955) (FR) with a total of 79 nominations, the physiologist and 1924 Nobel laureate Willem Einthoven (1860–1927) (NL) (31 nominations), the surgeon Alfred Blalock (1899–1964) (US) (29 nominations), the pharmacologist and 1936 Nobel laureate Otto Loewi (1873–1961) (DE, AT, US) (27 nominations) and the paediatric cardiologist Helen Taussig (1898–1986) (US) (24 nominations). The research of these scholars merely hints at the width of topics brought up by nominators ranging from the physiological and pathological basics to the diagnosis and (surgical) interventions of diseases such as heart malformation or hypertension*.*

**Conclusion:**

We argue that an analysis of Nobel Prize nominations can reconstruct important scientific trends within cardiovascular research during the first half of the twentieth century.

**Supplementary Information:**

The online version contains supplementary material available at 10.1007/s00392-021-01813-2.

## Introduction

Since 1901, the Nobel Prize has been awarded almost annually to scientists who, according to the prize jury, “have conferred the greatest benefit to mankind” (the key phrase in Alfred Nobel’s will) (https://www.nobelprize.org/alfred-nobel/alfred-nobels-will/, Access: 05/07/20). The award has become an unparalleled symbol of excellence within as well as outside the scientific community [1]. Receiving the Nobel Prize has usually resulted in a sudden increase in reputation for the laureate as well as in publicity regarding the respective field [2].

However, aside from the laureates, there are thousands of ‘unsung heroes’, i.e. nominees, whose names and nominations are under lock and key for 50 years before they are made publicly available. The current paper reconstructs which cardiovascular scientists were nominated between 1901 and 1953 and identifies the candidates who were considered runner-ups by the Nobel Committee—but failed shortly before the finish line to become a Nobel Prize laureate.

Previous analyses of cardiovascular research in the context of the Nobel Prize predominantly address the entirety of Nobel Prize laureates [3] or is limited to specific Nobel Prize laureates; for example, Willem Einthoven [4] (1860–1927) for the discovery of the electrocardiogram, Alexis Carrel [5] (1873–1944) for his work in the field of vascular surgery and transplantation, Werner Forssmann (1904–1979) [6] for his contribution to the development of heart catheterisation (1904–1979) and Joseph Erlanger [7] (1874–1965) for the discovery of different types of nerve fibres.

In light of several case studies of some of the unsuccessful nominees, such as the German internal specialist Hugo W. Knipping [8] (1895–1984), the German physiologist Hermann Rein [9] (1898–1953), and the US-American surgeon Alfred Blalock [10] (1899–1964), the current paper will contribute to the existing literature by identifying the "Nobel population “ of all nominees within the field of cardiovascular research. Such an approach has been carried out in other fields, most recently in pharmacology [11].

Compared to other disciplines within medicine, such as neurology (*American Neurological Association* founded in 1875), physiology (*American Physiological Society* founded in 1887) or surgery (*American College of Surgeons* founded in 1913), cardiovascular societies were late bloomers and only started to emerge worldwide during the first decades of the twentieth century. While the *German Cardiac Society* was founded as early as 1927, the *American College of Cardiology* was founded in 1949 and the *European Society of Cardiology* was established in 1950. Nevertheless, among the 5950 nominations (https://www.nobelprize.org/nomination/archive/, Access: 03/01/2020) which were assessed by the Nobel Committee for physiology or medicine from 1901 to 1953, several were from the field of cardiovascular research. Importantly, however, since most of the given research was interdisciplinary, the retrospective differentiation and affiliation of research areas and scientists is a meticulous task.

Given the aforementioned background, this article will discuss the following questions: Who were the most frequently proposed candidates in the field of cardiovascular research? What were the nominators’ main reasons for nomination, and which field of research was of particular interest to the prize jury? Which countries were key players within a Nobel context—and how did the list of countries change over time? To what extent do the Nobel nominations during the first half of the twentieth century reflect factors that might still be relevant for current prize contenders?

## Methods

Drawing from the Nobel nomination database (nobelprize.org) which contains 5950 nominations in the category of physiology or medicine from 1901 to 1953, we listed all scholars who were nominated due to their contributions to cardiovascular research. Our list includes the nominees who were proposed because of their research on the structure and function of the heart as well as the vasculature, the basics of blood flow and circulation, the diagnosis of cardiovascular diseases, as well as their conventional and surgical therapies. This includes several candidates who are (and were) primarily known for other research interests.

The selected time period results from the access to the digital archive of the Nobel Foundation, which to date only contains the nominations up to the year 1953. The rationales for the nominations, as listed in the Nobel nomination database (nobelprize.org), were summarised in prevailing cardiovascular research trends for each decade.

We subsequently compared all nominees with cardiovascular connection to the shortlisted candidates of the Nobel Committee to examine whether the award-worthiness of the nominees differed in the eyes of the nominators and the Nobel Committee. Additionally, we collected nomination letters and jury reports from individual candidates from the archives of the Nobel Committee in Sweden to illuminate characteristic lines of argumentation by nominators.

## Results

### An overview of research trends among all nominees: the first nominee Richard Thoma (1847–1923) and his work on arteriosclerosis

The first nominee with a connection to cardiovascular research was the German pathologist Richard Thoma (1847–1923) (Picture 1) for his work on arteriosclerosis. Born in Bonndorf in the Black Forest (Germany), Thoma studied medicine in Berlin and Heidelberg [12]. After his habilitation thesis about the movement of blood and lymph [13], he studied haemodynamics extensively [14].

**Picture 1:**
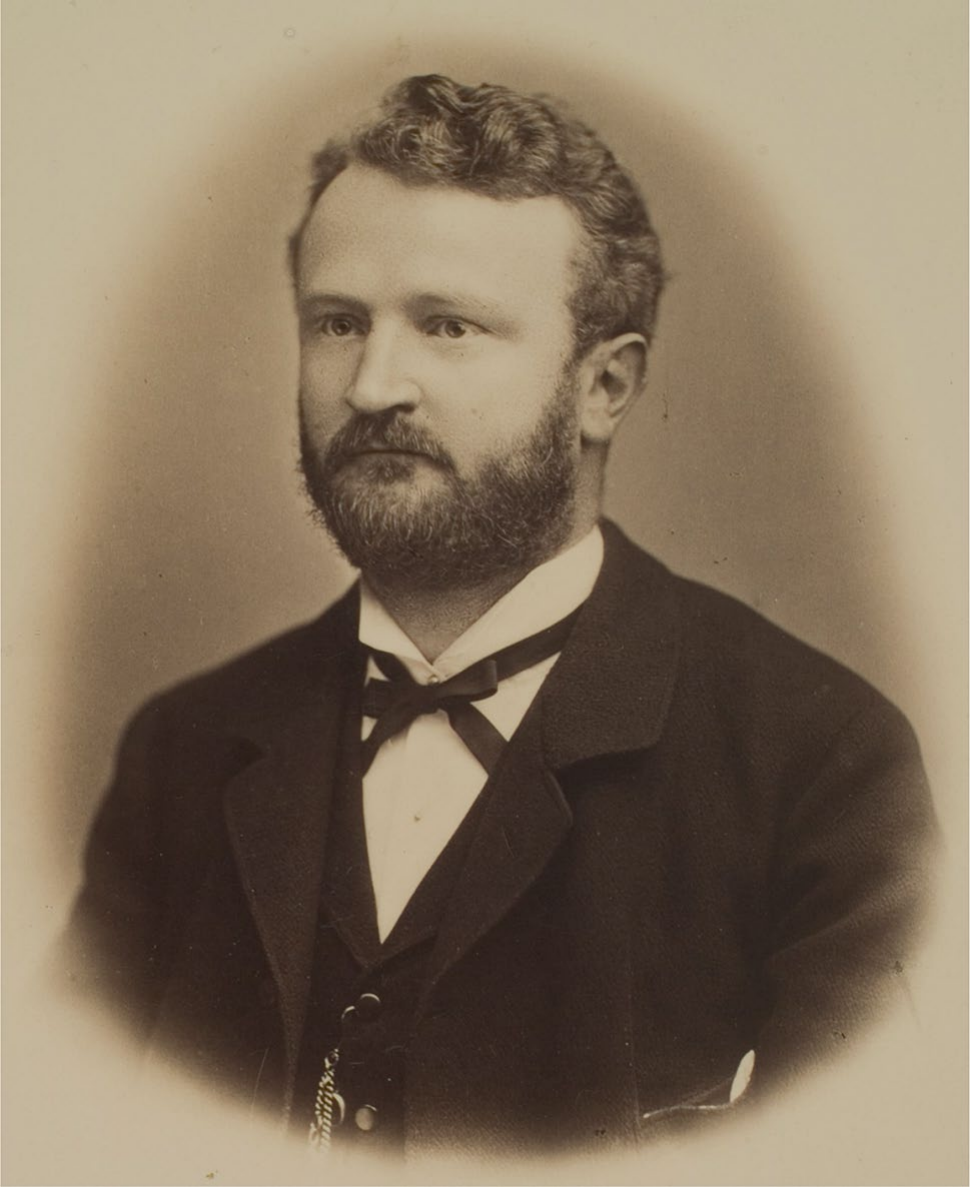
Richard Thoma (Universitätsbibliothek Heidelberg, CC BY-SA 4.0 < https://creativecommons.org/licenses/by-sa/4.0 > , via Wikimedia Commons)

In his letter of nomination, the Austrian internist Leopold Schrötter (1837–1908) stated:

*“I consider it my duty to point out the versatile work of an author who has been deliberately researching a disease for a number of years, which, through its widespread distribution and steady prevalence among the entire human race as well as its significant influence on mortality, is of extensive importance. I mean R. Thoma’s […] research on arteriosclerosis.”*

(Nobel Archive, Nomination of Richard Thoma by Leopold Schrötter, 24th of January 1901. Translated from German)

This prelude to a disease which is so prevalent today remains an exception as cardiovascular nominations during the first two decades of the twentieth century focused primarily on the innervation of the heart, the basics of blood circulation as well as progress in diagnostics (compare longlist, supplement). Other research concerning cardiovascular diseases primarily included studies on arterial hypertension in addition to research addressing arteriosclerosis. Representatives of these research areas were, for example, the Russian Nikolaus Anitschkov (1885–1964), the US-American Harry Goldblatt (1891–1977), and the US-American Irvine Page (1901–1999). With their nominations in the late 1930s to early 1940s, these researchers are found much later in the observed time period.

As shown in Table 1, a sub-group of cardiovascular research, on which most of the nominations are based, can be identified for almost every decade. Thus, we are able to pinpoint research trends regarding subject areas that particularly attracted the nominators' interests during the first five decades of the twentieth century. These research trends reveal a development from basic research to clinical practice, ranging from the influence of hormones and electrolytes on the heart muscle to surgical therapies of heart malformations.

**Table 1:** Cardiovascular research trends 1901–1950

Decade	Research trends
1901–1920	Principles of heart innervation and blood circulation, developments in diagnostics
1921–1930	Heart mechanism (electrolytes and hormones)
1931–1940	Regulation of blood circulation and blood pressure
1941–1950	Surgical therapies of heart malformations and diseases

### What is of interest over time?

Between the nomination and the awarding of the Nobel Prize, it is the task of the Nobel Committee to shortlist potential laureates. Today, the Nobel Committee consists of six professors for medicine at the Karolinska Institute in Solna, Sweden, who are appointed for a period of 3 years. The committee proposes one–three candidates from the pool of all nominees, while the Nobel Assembly (fifty elected professors at the Karolinska Institute) makes the final decision. Although both the Nobel Committee and the Nobel Assembly are situated at the Karolinska Institute, they are not a part of the Institute but of the Nobel Foundation.

Table 2 shows all scholars who were nominated because of their contribution to cardiovascular research between 1901 and 1953 and were taken into closer consideration by the Nobel Committee during the same year. While, in general, we can see an increasing number of shortlisted cardiovascular candidates over time, we can also observe that the attention of the Nobel Committee did not focus on scholars who investigated the same cardiovascular research trend. Instead, the interest of the committee largely complied with the general consensus on a variety of topics that prevailed each decade from 1900 to the 1950s.

**Table 2:** Shortlisted nominees with cardiovascular connections 1901–1953

Decade	Year of shortlisting	Shortlist candidate	Research topic
1901–1910	1903	Étienne-Jules Marey (FR)	Functional diagnostics and blood circulation
1911–1920	1912	Alexis Carrel (FR)	Transplantation of blood vessels (Annotation: Awarded with a Nobel Prize in 1912 “in recognition of his work on vascular suture and the transplantation of blood vessels and organs.”)
	1913, 1914, 1924	Willem Einthoven (NL)	Functional diagnostics and heart physiology (Annotation: Awarded with a Nobel Prize in 1924 “for his discovery of the mechanism of the electrocardiogram”)
	1919, 1920	August Krogh (DK)	Various reasons for nomination (Annotation: Awarded with a Nobel Prize in 1920 "for his discovery of the capillary motor regulating mechanism”)
	1920	Sir James Mackenzie (GB)	Heart physiology and -diseases
1921–1930	1924	Sir Thomas Lewis (GB)	Heart auricles (fibrillation and flutter)
	1927, 1928	Otto Loewi (AT)	Heart hormones and chemical transmission of nerve impulses in the heart
	1927	Ludwig Haberlandt (AT)	Heart hormones
	1928	Otto Frank (DE)	Variations in haemodynamics
1931–1940	1934	Heinrich Hering (DE)	Blood pressure restraints
	1934, 1936	Corneille Heymans (BE)	Regulation of blood pressure
	1936, 1943	René Leriche (FR)	Arteriectomy
1941–1950	1941	Harry Goldblatt (US)	Hypertension and renal ischemia
	1941	Irvine Page (US)	Arterial hypertension
	1941	Arthur Stoll (CH)	Heart glycosides
	1947, 1949	Alfred Blalock (US)	Surgery of heart malformations
	1947, 1949	Helen Taussig (US)	Surgery of heart malformations
	1949	Norbert Goorghmatigh (BE)	Hypertension
	1949	Robert Gross (US)	Surgery of heart malformations
	1949	Claude Beck (US)	Surgery of heart malformations

During the first decade of the twentieth century, the attention of the Nobel Committee was only drawn to the French physiologist Étienne-Jules Marey (1830–1904) (Picture 2). Known as a pioneer in circulatory physiology [15] and blood pressure measurement [16], he was particularly praised for his experiments on intracardiac blood pressure measurement in horses together with the French veterinarian Auguste Chauveau (1827–1917) [17, 18]. Étienne-Jules Marey was nominated 3 times in 1903 and 1904. His nominators, who came from France and Austria, alluded to both his research on graphic and photographic diagnostics of the pulse rate as well as his work on blood pressure circulation from 1880. Given these reasons for nomination, Marey fitted well into the research trends of his time on both counts in a Nobel context (Table 1).

**Picture 2:**
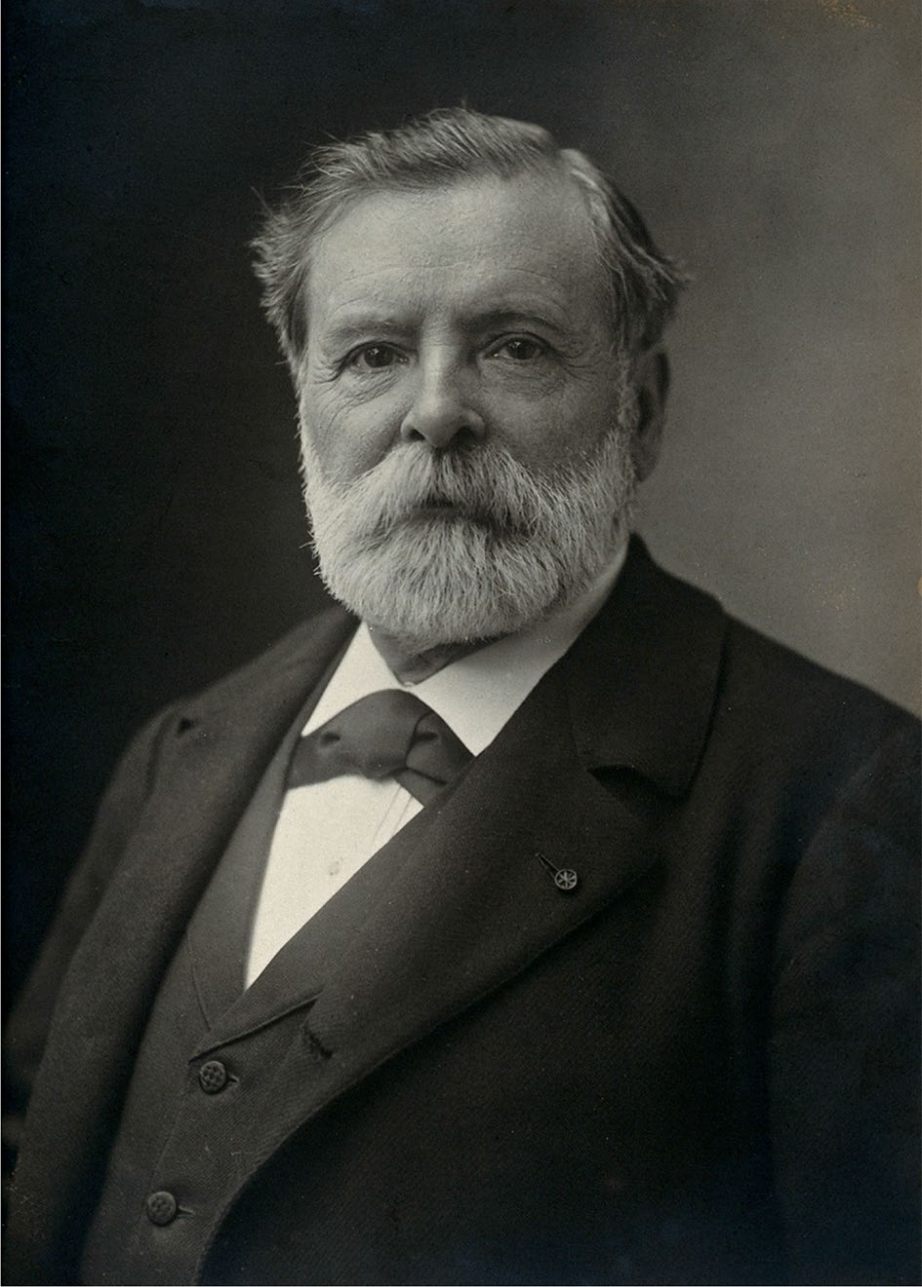
Étienne-Jules Marey (Photograph by Nadar, CC BY 4.0 < https://creativecommons.org/licenses/by/4.0 > , via Wikimedia Commons)

However, Marey’s nomination was followed by a 10-year period during which no cardiovascular researcher was considered more closely, while the second decade yielded three shortlisted scholars who were awarded a Nobel Prize in 1912, 1920, and 1924. As a consequence, cardiovascular research experienced its first peak of international recognition in a Nobel Prize context with the nominations of Alexis Carrel, August Krogh (1874–1949) and Willem Einthoven (Table 2).

Consequently, the number of shortlisted cardiovascular scholars among all shortlisted candidates grew continuously. As the shortlisted candidates were nominated for various cardiovascular research topics, the increasing number of these nominees shows a generally increasing interest in cardiovascular research on the part of the Nobel Committee.

### Research trends and hotspots: from Western/Central Europe to the United States

Richard Thoma, the first cardiovascular Nobel nominee in 1901, can be seen as an example of the geographic focus that would pervade the nominations in the following decades. We found an 80 percent share of Western and Central European candidates, not only among the nominees, who were primarily scholars from Germany and France, but also among their nominators. Central Europe, during the first four decades of the twentieth century, was, specifically in a Nobel Prize context, the hotspot of cardiovascular science and research. Consequently, the most frequently nominated researchers with cardiovascular connections from 1901 to 1940 were the French surgeon René Leriche (1879–1955) with a total of 79 nominations, the Dutch 1924 Nobel laureate Willem Einthoven with 31 nominations and the German pharmacologist and 1936 Nobel laureate Otto Loewi (1873–1961) with a total of 27 nominations.

As of today, the top candidate Leriche (Picture 3) is considered to be one of the pioneers of vascular surgery [19]. Serving as an example is the eponym *Leriche syndrome* which refers to the symptoms of an obstruction in the area of the pelvic artery branching [20]. Among his overall 79 nominations, Leriche was nominated in 1936 by his French colleague and 1928 Nobel Prize Laureate Charles Jules Henri Nicolle (1866–1936) for his discovery of the physiological and therapeutic effects of resecting obliterated arteries. Furthermore, Leriche was proposed because of his work on sympathetic surgery, especially periarterial sympathectomy, as well as his wartime examinations on the physiology and treatment of pain.

**Picture 3:**
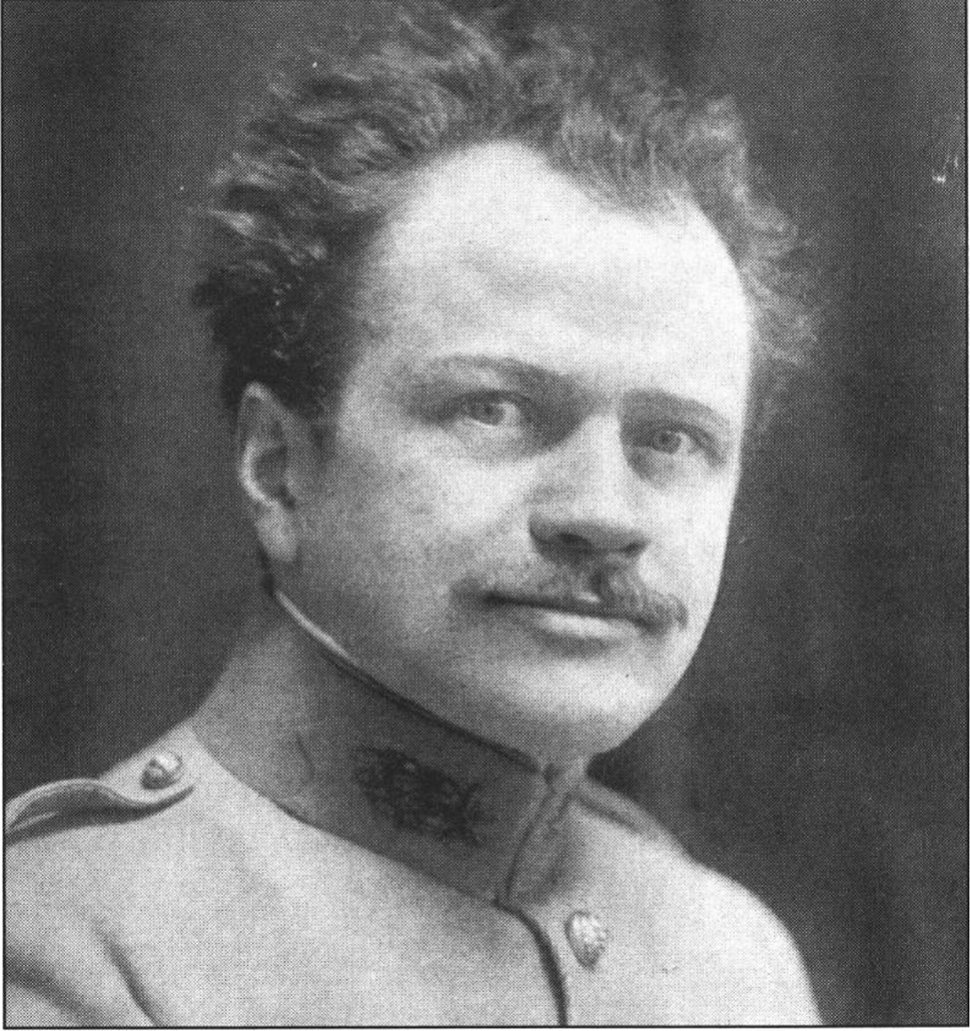
René Leriche 1915 (Unkown Author, Public domain, via Wikimedia Commons)

**Picture 4:**
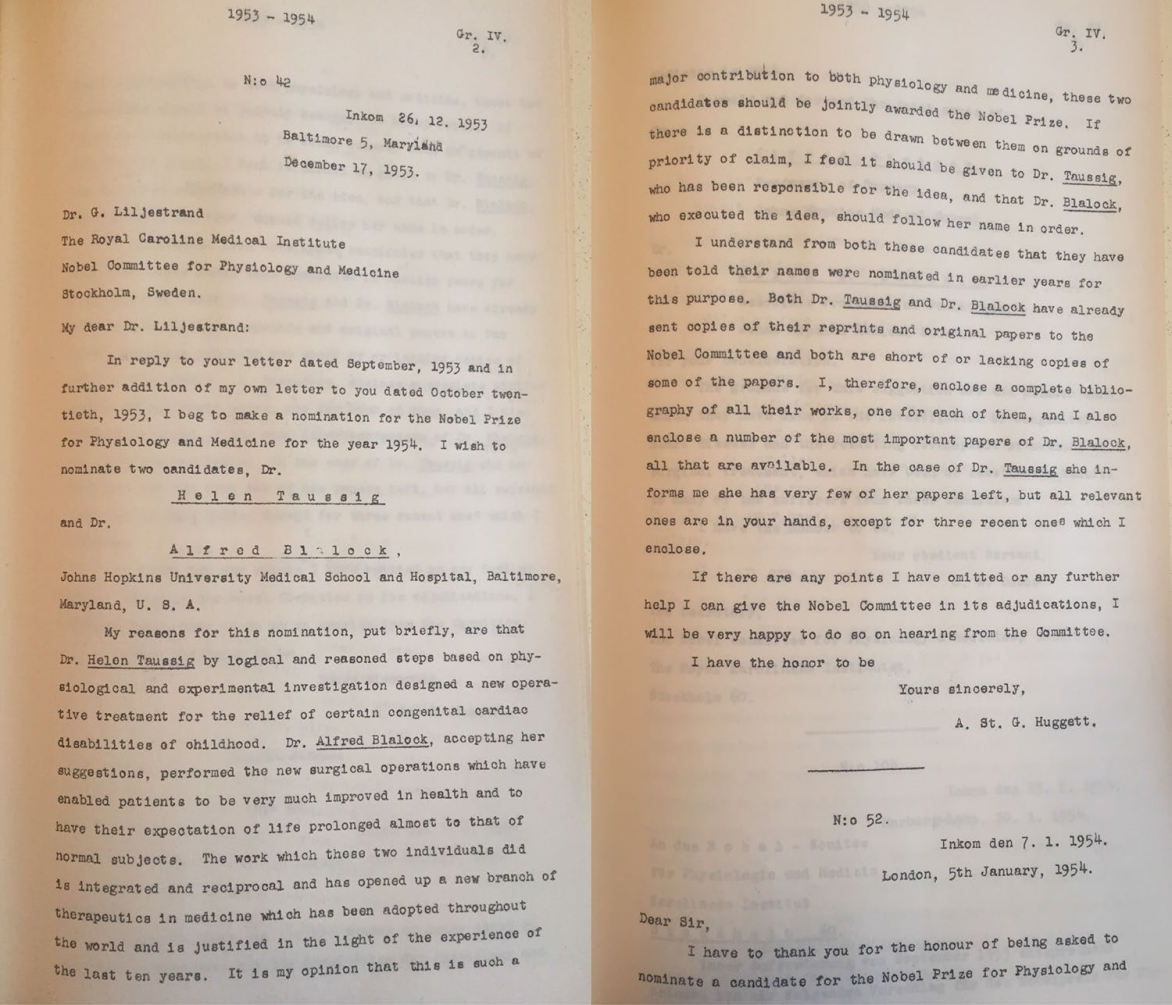
Archive of the Nobel Committee, Nomination for Helen Taussig and Alfred Blalock, written by A.St.G. Huggett. December 17, 1953

The ‘Nobel European era’ lasted until the beginning of the Second World War. It was succeeded by scholars in the United States who made up the lion’s share (about 60%) of the nominees in the following years. Aside from economic factors, English as the now-established language of research was a decisive advantage [21]. The Nobel nominations of the 1940s mainly revolved around a group of US scholars who studied the surgical treatment of cardiac abnormalities. Out of this group, the surgeon Alfred Blalock (1899–1964) with a total of 29 nominations and the paediatric cardiologist Helen B. Taussig (1898–1986) with 24 nominations complete our “Top 5” of most frequently nominated cardiovascular scholars between 1901 and 1953.

### In trend and yet a countertrend? The Nobel nominations for the first female cardiovascular nominee Helen B. Taussig

In 1947, as a US-American and an example of cardio-surgical nominations, Helen Taussig was the first female cardiovascular researcher to be nominated for a Nobel Prize.

Born on May 24, 1898 in Cambridge, Massachusetts, Helen Taussig studied at Radcliffe College for two years before joining the University of California at Berkeley. From 1923 to 1927, she attended the Johns Hopkins School of Medicine. Afterwards, on her way to becoming a paediatrician, she received a fellowship in the medical cardiovascular division. Here, she found a confederate in Alfred Blalock. Together, they developed the technique of a subclavian anastomosis to the pulmonary artery [22], which proved successful on November 29, 1944. Due to her success, Helen Taussig became associate professor of paediatrics in 1946. In 1965, again as the first woman, she was elected president of the American Heart Association [23] (Picture 4).

Helen Taussig was nominated 24 times from 1947 to 1953 by nominators from, predominantly, the United States but also by the internal specialist Hugo Knipping [8] (1895–1984) from Germany. Knipping stated in his nomination letter for the 1952 prize:

*“The enormous development of cardiac surgery can be attributed to the two researchers Alfred Blalock and Helen Taussig, who were able to perform the first successful operation of a cardiac vitium—the pulmonary stenosis—at the Johns Hopkins Hospital in Baltimore around 1945. This intervention, ever since known as the “blue baby operation”, was not only a unique medical feat for surgery, but also stimulated the internal heart clinic, especially by providing the impulse for intracardiac diagnostic methods that were subsequently developed until perfection and whose therapeutic purpose lies in the preparation for operative therapy. In both researchers we honour the scientist, discoverer and doctor.”*

(Nobel Archive, Nomination of Alfred Blalock and Helen Taussig by Hugo Knipping, 12th of December 1951. Translated from German)

Shortly after the first nominations for Blalock and Taussig, the Nobel Committee requested in-depth expert opinions on them, starting with a brief investigation in 1947 and then more detailed reports in 1949, 1954, and 1956. Although the experts, for example, John Hellström (1890–1965), Professor of Surgery at the Karolinska Institute, considered Blalock and Taussig's research to be groundbreaking for future cardio-surgical developments, the experts still found it difficult to identify only a few pioneers from the large group of cardiac surgeons. Moreover, as the time span since the development of Blalock’s and Taussig’s technique increased, the operative method became increasingly refined. The rapid development of cardiosurgical techniques had the consequence that Blalock's and Taussig’s achievement seemed outdated again.

## Discussion

A look at the previous laureates (1901–2020) shows that several topics with ties to cardiovascular research were celebrated throughout the decades (Table 3), ranging from the electrocardiogram (Einthoven in 1924, Picture 5) to discoveries concerning nitric oxide as a signalling molecule in the cardiovascular system (Furchgott, Murad, Ignarro in 1998). However, the more recent prospects were not that promising: during the last 20 years, no cardiovascular scientist has received the golden Nobel medal. We argue that Nobel Prize nominations not only can reconstruct important scientific trends within cardiovascular research over time, but also that some aspects brought up in nominations during the first half of the twentieth century are still relevant today. In the following, we will highlight three of them.

**Table 3:** Nobel Prize Laureates in physiology or medicine with cardiovascular connections 1901–2020

Year	Nobel laureate(s)	Official prize motivation
1912	Alexis Carrel (1873–1944)	“In recognition of his work on vascular suture and the transplantation of blood vessels and organs”
1920	August Krogh (1874–1949)	“For his discovery of the capillary motor regulating mechanism”
1924	Willem Einthoven (1860–1927)	“For his discovery of the mechanism of the electrocardiogram”
1956	Werner Forssmann (1904- 1979), Andre Cournand (1895–1988), Dickinson W. Richards (1895–1973)	“For their discoveries concerning heart catheterization and pathological changes in the circulatory system”
1964	Konrad Bloch (1912–2000), Feodor Lynen (1911–1979)	“For their discoveries concerning the mechanism and regulation of the cholesterol and fatty acid metabolism”
1982	Bengt Samuelsson (1934), Sune Bergström (1916–2004), John Vane (1927–2004)	“For their discoveries concerning prostaglandins and related biologically active substances”
1985	Michael Brown (*1941), Joseph Goldstein (*1940)	“For their discoveries concerning the regulation of cholesterol metabolism”
1988	James Black (1924–2010), Gertrude Elion (1918–1999), George Hitchings (1905–1998)	“For their discoveries of important principles for drug treatment” (Beta Blockers)
1998	Robert Furchgott (1916–2009), Ferid Murad (1936), Louis Ignarro (1941)	“For their discoveries concerning nitric oxide as a signalling molecule in the cardiovascular system”

**Picture 5:**
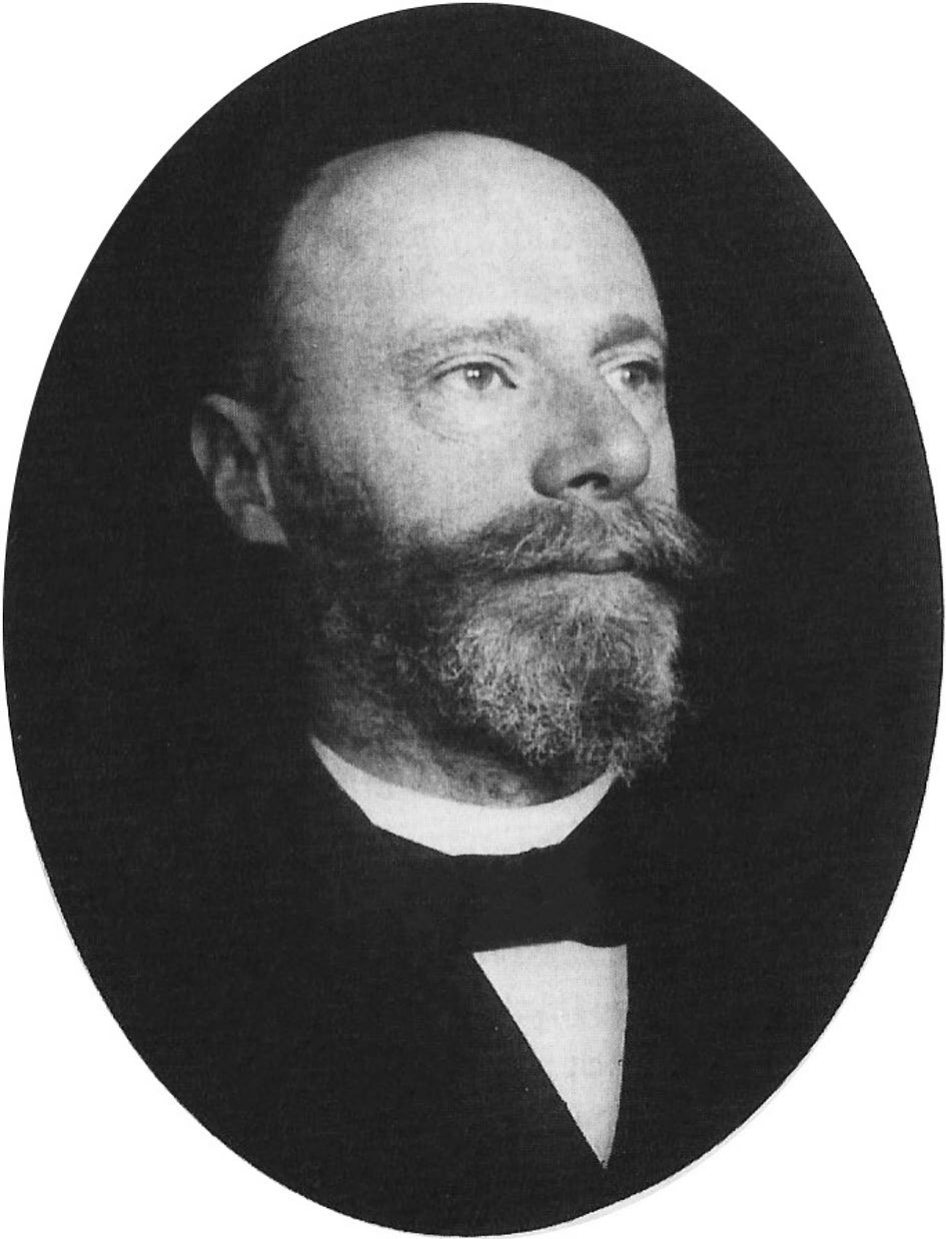
Willem Einthoven 1906 (Unkown Author, Public domain, via Wikimedia Commons)

### Ever-growing research teams: Is the “lone discoverer” a residue from days gone by?

As mentioned above, Blalock and Taussig accumulated more than 50 nominations for their developments in the surgical treatment of the tetralogy of Fallot. Yet, additionally, numerous other cardiac surgeons were nominated in the 1940s and 1950s making it a delicate task for the Nobel Committee to identify individual pioneers deserving of the Nobel Prize. In the end, the Nobel Committee could not agree on one to three scholar(s) as being the most important. The scientific community in the field was simply judged as too big and the scientific priority disputes nearly impossible to handle. In light of the current trend to carry out research in large consortia, this risk of not receiving a prize due to huge research teams is even more relevant today [24].

The requirement that only one to three individual scientists may be awarded the Nobel Prize at the same time has, therefore, been much discussed in recent years. In 1998, sociologist and historian Elisabeth Crawford, one of the first to gain access to the Nobel Archives, stated “the idea of the lone discoverer lingers on as a myth” as nowadays scientific research is of a “group-orientated nature” [25]. The calls of individual scientists to reform the Nobel Prize in this regard [26, 27] have so far been unheard.

In 2019, however, Wu et al. showed that despite the trend towards ever-growing research teams, larger research teams mainly continue existing work, while smaller research teams succeed in making the most pioneering advances [28]. Thus, while the continuation of existing and recognised work may have some advantages in obtaining funding or publications and improving patient care, Alfred Nobel's call to reward the most groundbreaking discovery in medicine will probably, therefore, rather continue to be fulfilled by smaller research teams.

### The old Nobel dilemma: clinical versus basic research

While in the early days of Nobel history several prizes went to clinical researchers, many scientists lament the overwhelming majority of basic researchers who have been awarded a Nobel Prize in recent decades [29]. A glance at the Nobel Prizes awarded in the last ten years, for example, reveals a focus on biological and biochemical mechanisms at the cellular level. Recognition for clinical advances can also be found in early cardiovascular research, such as the award for the electrocardiogram, vascular surgery (Carrel in 1912, Picture 6), and cardiac catheterisation (Forssmann in 1956, Picture 7). But in cardiovascular research, too, basic research has dominated the prize field in the second half of the twentieth century.

**Picture 6:**
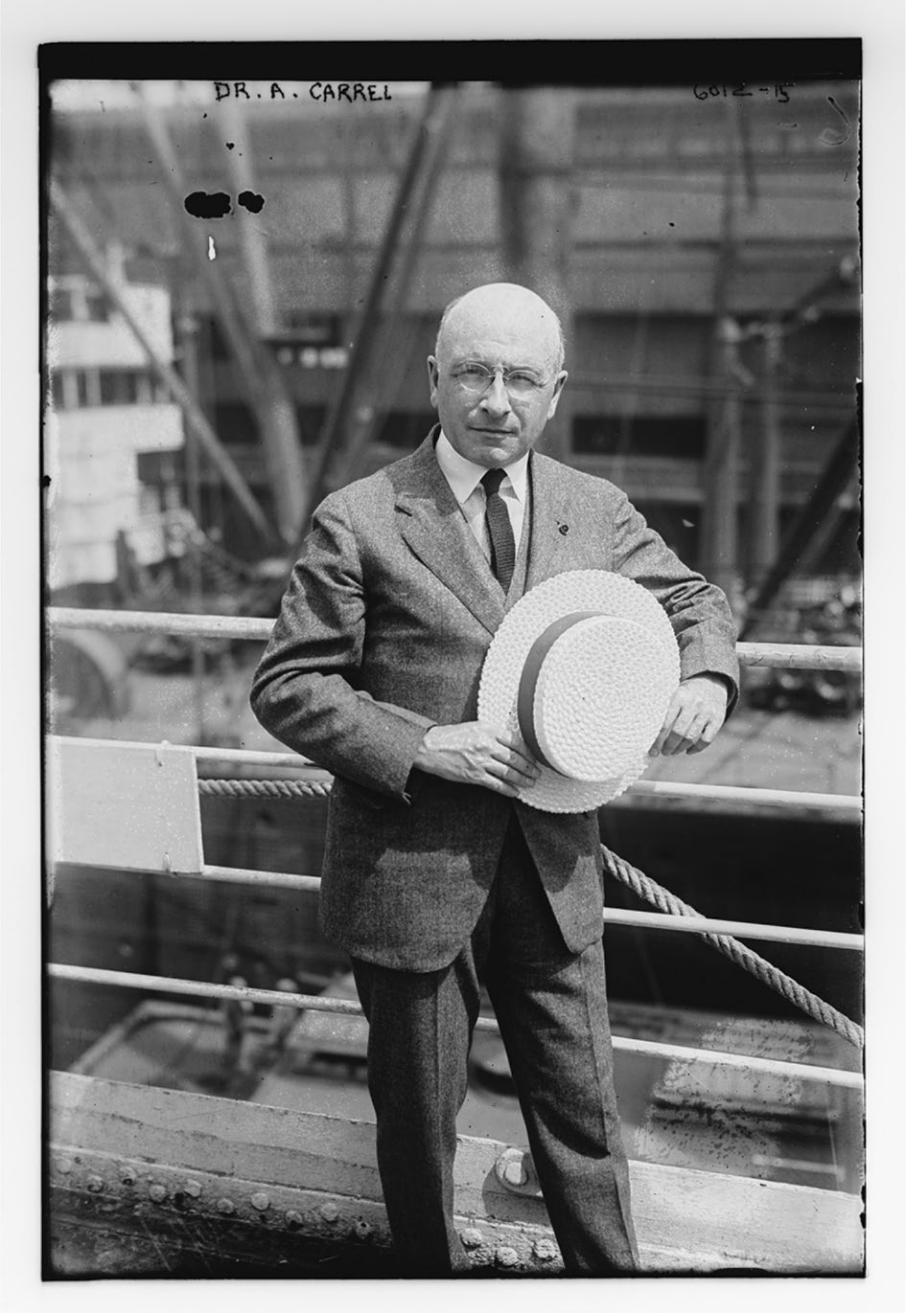
Alexis Carrel (Bain News Service, Public domain, via Wikimedia Commons)

**Picture 7:**
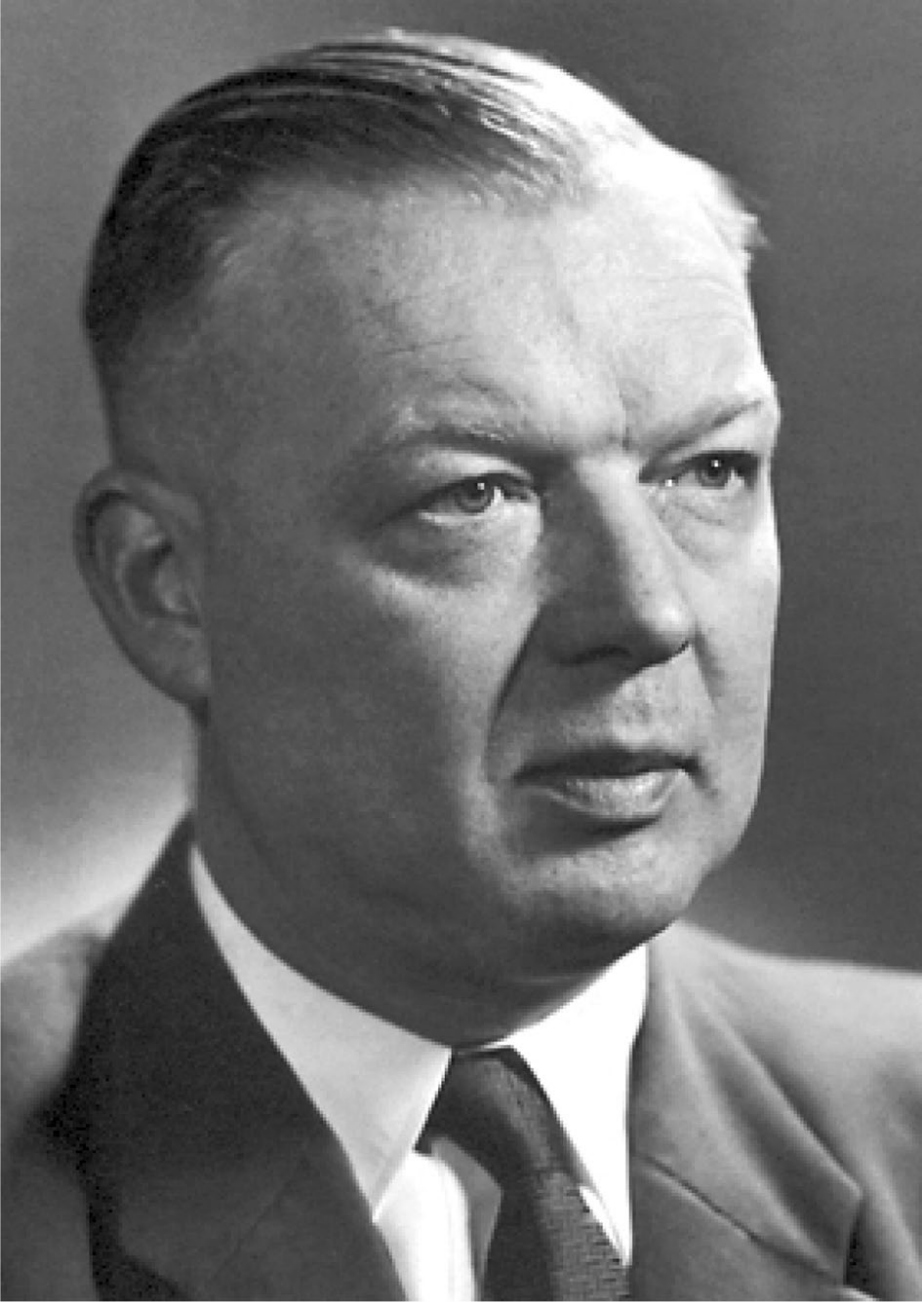
Werner Forssmann (Unkown Author, Public domain, via Wikimedia Commons)

In particular, the rapid development of cardiovascular drugs in the twentieth century is a category of basic research that has been honoured several times with a Nobel Prize. For instance, the Nobel Prize of 1988 was awarded to James Black (1924–2010) (Picture 8) (whose individual award citation refers to the discovery of propranolol (https://www.nobelprize.org/prizes/medicine/1988/black/facts/, Access: 13/11/20)), Gertrude Elion (1918–1999) and George Hitchings (1905–1998) (Picture 9) “for their discoveries of important principles for drug treatment”. In addition, the Nobel Prizes of 1964, 1982 (Captopril), 1985, and 1998 (Nitrates) were also awarded for discoveries in the field of cardiovascular research which led to new drug therapies. The cholesterol metabolism, for example, which served as motivation for the Nobel Prize twice in both 1964 and 1985, led not only to a deeper understanding of the pathology of cardiovascular diseases but also to the development of cholesterol-lowering drugs such as statins. As the Nobel archives become available for the 1970s, it will become possible to see how the committee responded to other wide-reaching discoveries in the field, such as heart transplantation, coronary artery grafting in cardiac surgery, and angioplasty of coronary arteries [30–33]. Given the 50-year embargo on Nobel nominations and evaluations, such evaluation processes are kept a cliff-hanging secret [34]. The background of a prize decision and the reasons for or against a candidate are, therefore, only speculations. That being said, the current composition of the Nobel Committee reveals that neither clinicians nor cardiovascular researchers on board dorm part of the committee, which could put a potential barrier in the way of cardiovascular researchers.

**Picture 8:**
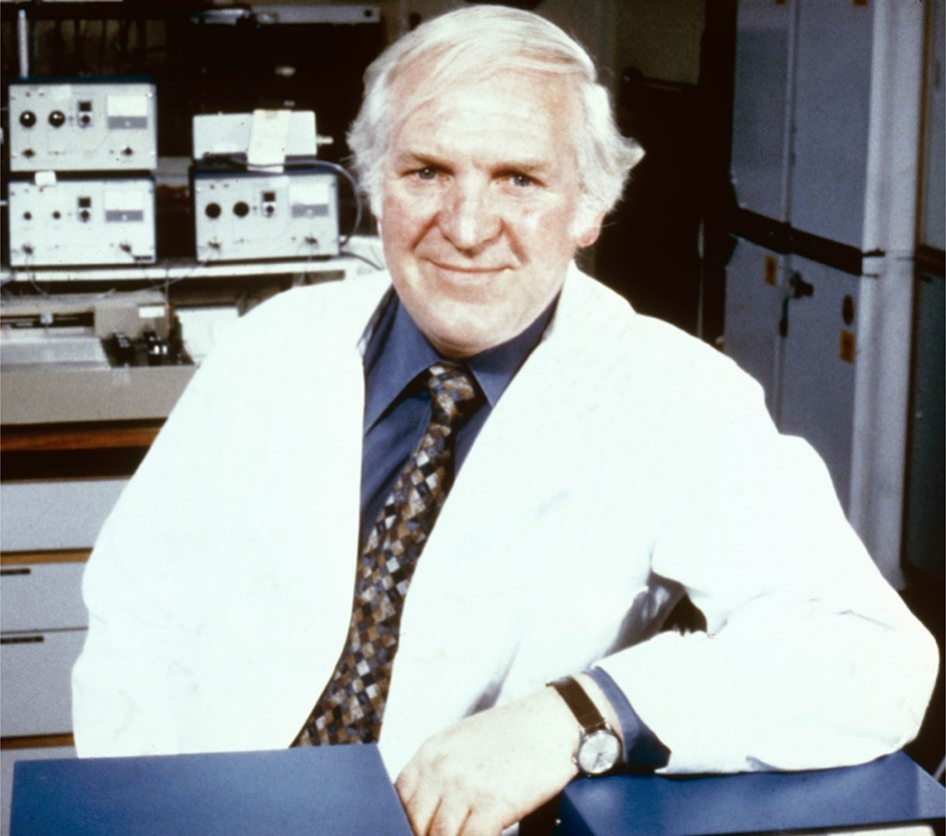
James Black (Unknown author, CC BY 4.0 < https://creativecommons.org/licenses/by/4.0 > , via Wikimedia Commons)

**Picture 9:**
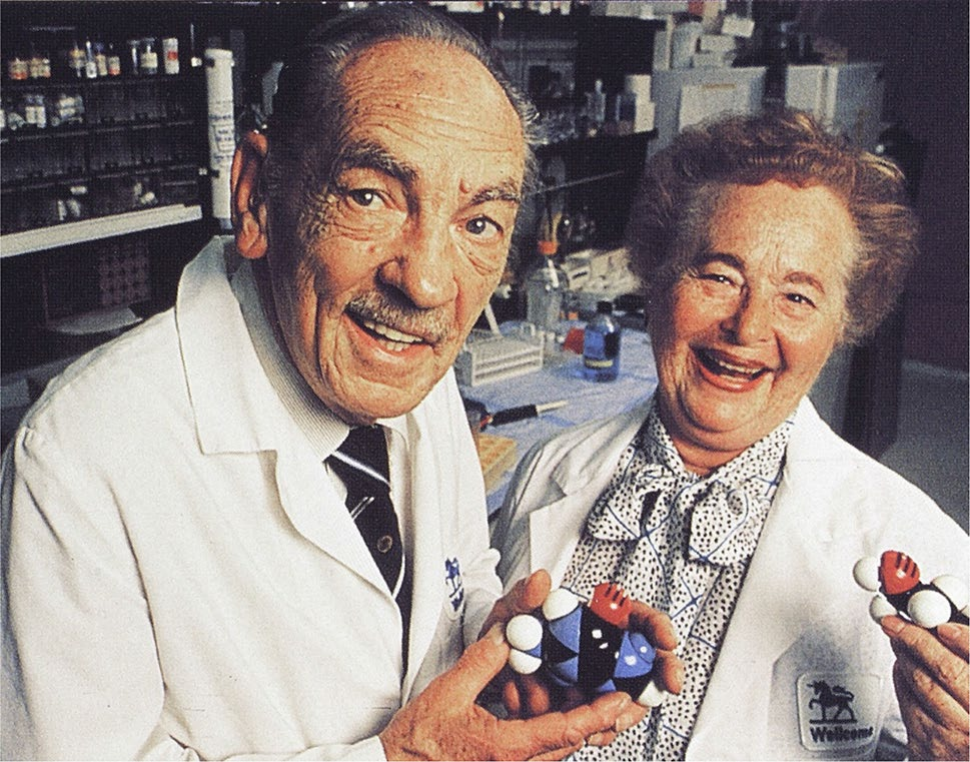
George Hitchings and Gertrude Elion (Unknown author, CC BY 4.0 < https://creativecommons.org/licenses/by/4.0 > , via Wikimedia Commons)

## Beyond Nobel: can other prestigious prizes offer any insights?

 There are several renowned awards that honour top-class scientists every year. The striking consensus of laureates from the US-American Lasker Award or the Canada Gairdner Award to those of the Nobel Prize led to their image as a type of “pre-Nobel Prize”. In 2013, Siqi Ye et al. statistically demonstrated that in the examined period from 1983 to 2012, almost 70% of the Nobel laureates previously received a Gairdner Award and almost 60% of the Nobel laureates had previously won a Lasker Award. The time span between winning a Gairdner award and receiving the Nobel Prize was in most cases between 5 and 10 years [35]. As a flicker of hope, both the Lasker and Gairdner prize juries have recognised cardiovascular laureates over the last 20 years, such as Akira Endo (b. 1933), Salim Yusuf (b. 1952) or Alain Carpentier (b. 1933) and Albert Starr (b. 1926). Even if these award-winners may not become the next Nobel laureates, they show that cardiovascular research still is an award-winning field.

## Supplementary Information

Below is the link to the electronic supplementary material.

Supplementary file1 (DOCX 28 KB)

## Data Availability

Data available within the article or its supplementary materials.
