# Supplementary material for: Who is who in cardiovascular research? What a review of Nobel Prize nominations reveals about scientific trends
Source: Clin Res Cardiol. 2021 Mar 6;110(12):1861–70. doi: 10.1007/s00392-021-01813-2 (PMC8639567; doi:10.1007/s00392-021-01813-2)
Supplement: Supplementary file 1 — Supplementary file1 (DOCX 28 KB) [file 392_2021_1813_MOESM1_ESM.docx]

| Cardiovascular Nominees 1901-1953 | | | | | |
| --- | --- | --- | --- | --- | --- |
| **Nominee with cardiovascular connection** | **Nomination from … to** | **Number of Nominations** | **Nominator(s)** | **Cause of Nomination** | **Shortlist Candidate** |
| **Richard Thoma (DE)** | 1901 | 1 | Leopold Schrötter (AT) | Work on arteriosclerosis |  |
| **Theodor Wilhelm Engelmann (DE)** | 1901-1906 | 3 | K.J. Gerhardt (DE), Theodor Ziehen (NL), Cornelius Adrians Pekelharing (NL) | Physiology of the nervous system in relation to the function of the heart; Work on the myogenic origin of the action of the heart |  |
| **Étienne-Jules Marey (FR)** | 1903-1904 | 3 | Alexander Rollett (AT), Marius Tscherning (FR), Charles Robert Richet (FR) | Development of graphic and chronophotographic methods to study, e.g. (…) pulse rate(…); Work on blood circulation (1880) | 1903 |
| **Auguste Chauveau (FR)** | 1903-1916 | 24 | C. Sigalas (FR) et al. | Work in experimental pathology, physiology (electrophysiology and mechanics of the circulatory system) and metabolism. |  |
| **Giulio Vassale (IT)** | 1905-1911 | 10 | Vittorio Mibelli (IT) et al. | General atony and arterial hypotension |  |
| **Jean Dogiel (RU)** | 1907 | 1 | A. N. Kasem Beck (RU) | Physiological and anatomical studies of the organs of sight and hearing, as well as the heart and blood circulation. |  |
| **Oskar Langendorff (DE)** | 1908 | 1 | Rudolf Kobert (DE) | Studies of the heart of warm-blooded animals, and methods for prolonging life of the heart after removal from the body. |  |
| **Henri Huchard (FR)** | 1910 | 1 | Albert Robin (FR) | Work on the blood circulation organs |  |
| **Wilhelm Erb (DE)** | 1910-1914 | 2 | N. Ordner (AT), H. Ordner (AT) | Work on electro diagnostics |  |
| **Willem Einthoven (NL)** | 1911-1924 | 31 | Edouard Laguesse (BE), Alexandre Jarotzkijet (EE) et. al. | Work on the physiology of the heart; Work on the electrocardiogram. | 1913 1914 1924 |
| **Alexis Carrel (FR)** | 1912-1913 | 3 | Charles Bouchard (FR), William Keen (US), T. Rumpf (DE) | Work on the transplantaion of blood vessels and organs | 1912 |
| **Sir Thomas Lauder Brunton (GB)** | 1913 | 1 | Emanoil Riegler (RO) | No Motivation (Annotation: primarily known for the introduction of amyl nitrite in the treatment of angina pectoris) |  |
| **Sir James Mackenzie (GB)** | 1913-1921 | 4 | Edouard Laguesse (BE), Charles Fedeli (IT), Sir Clifford Allbutt (GB), Sir Ronald Ross (GB) | Work on the physiology of the heart; Work on the diseases of the heart and the physiology of the heart muscle | 1920 |
| **Ivan M Dogiel (RU)** | 1914 | 1 | L. Darkchevitch (RU) | Work on the innervation of the heart. |  |
| **Ludwig Aschoff (DE)** | 1917-1934 | 9 | M. Matthes (DE), O. von Schjerning, (DE), Otto Naegeli (CH), N. Ortner (AT), A. Policard (FR), Walther Fischer (DE), H. Eppinger (DE), Franz Orsos (HU) | Clinical pathological studies, work on general pathology and the heart; Work on arteriosclerosis, inflammation |  |
| **Ludolf von Krehl (DE)** | 1917-1932 | 2 | M. Matthes (DE), E. von Romberg (DE) | Internal medicine issues from the biological view of pathophysiology.  (Annotation: Mainly diseases of the heart muscle and nervous heart diseases) |  |
| **August Krogh (DE)** | 1919-1920 | 3 | J. Lindhard (DK), Nathan Zuntz (DE), Johan Johansson (SE) | Work on respiratory methods, gas exchange in the lungs, minute volume of the heart, micro gas analysis and the effect of abnormal nutritional conditions on metabolism. | 1919  1920 |
| **Joseph Barcroft (GB)** | 1919-1943 | 13 | Nathan Zuntz (DE) et al. | Work on blood distribution in higher organisms; Work on the physiology of respiration, hemoglobin and circulation. | 1919  1936 |
| **Robert Tigerstedt (FI)** | 1919-1923 | 4 | Nathan Zuntz (DE), Ali Krogius (FI), T.W. Tallqvist (FI), W. Pipping (FI) | The work "Die Physiologie des Kreislaufes" |  |
| **John S. Haldane (GB)** | 1920-1935 | 5 | Yandell Henderson (US), Paul Hoffmann (DE), A. Burkitt (AU), Whitridge Davies (AU), Claude Witherington Stump (AU) | Work on blood gases, circulation and respiration |  |
| **Friedrich Martius (DE)** | 1924 | 1 | Hans Curschmann (DE) | The foundation of clinical constitutional research (Annotation:Cardiac functional diagnostics: registration of the apex beat and assignment of the heart sounds) |  |
| **Sir Thomas Lewis (GB)** | 1924-1940 | 7 | Sir Archibald Hill (GB), Francis R. Fraser (GB), Geo Gask (GB), T. Addis (US), A. Clerk-Kennedy (GB), William Gerrard (HK), John Ryle (GB) | Work on the mechanism of the heart and on the mechanism of such disorders as Auricular flutter and Auricular fibrillation; Work with the electrocardiograph, which has been instrumental in making clear many of the problems concerning diseases of the heart. | 1924 |
| **Filippo Bottazzi (IT)** | 1925-1941 | 6 | Carlo Foà (IT), Giovanni Gallerani (IT), Giuseppe Verrotti (IT), Giuseppe Favaro (IT), Alberto Aggazzotti (IT), Ruggero Balli (IT) | Work on the function of the spleen, the effect of potassium on the heart and the contraction of the sarcoplasm. |  |
| **Sir Henry Hallett Dale (GB)** | 1926-1936 | 21 | M. Ide (BE) et al. | Work on adrenelin, ergotamine and histamine. Studies of the capillary system | 1936 |
| **Otto Loewi (AT)** | 1927-1936 | 27 | E. T. von Brücke (AT) et al. | Discovery of an hormonal system controlling action of the heart. | 1927 1928 |
| **Ludwig Haberlandt (AT)** | 1927 | 2 | Felix Sieglbauer (AT), Konrad Staunig (AT) | Hormonal control of the heart muscle, especially in his work "Das Hormon der Herzbewegung" | 1927 |
| **Otto Frank (DE)** | 1928-1937 | 8 | F. Proell (DE), W. Meisner (DE), H. Straub (DE), W. Schönfeld (DE), Otto Krummacher (DE), R. Rosemann (DE), W. Trendelenburg (DE), K. Bürker (DE) | Theory behind registration instruments. Mathematical and physical calculations for studying problems in haemodynamics such as variations in pressure, volume and flow rate in different areas of the circulatory system. | 1928 |
| **Antonio C. de Abreu Freire Egas Moniz (PT)** | 1928-1950 | 18 | P. Azevedo Neves (PT) | Work on encephalography on arteries and veins and the method´s use in the determination of blood speed.  (Annotation: 1949 Nobel Laureat “for his discovery of the therapeutic value of leucotomy in certain psychoses” - hereof nominations starting in 1937) |  |
| **Victor Pachon (FR)** | 1928 | 9 | Jacques Carles (FR), William Dubreuilh (FR), L. Beille (FR), C. Sigalas (FR), H. Mandoul (FR), G. Petges (FR), J. Guyot (FR), R. Cruchet (FR), Henri Verger (FR) | Determination of the minimal (diastolic) blood pressure, anticoagulatory effect of the liver, role of calcium in heart function, extirpation of the stomach and studies of stomach surgery, and studies of heart function. |  |
| **Friedrich Kraus (DE)** | 1929 | 1 | A. N. Rubel (RU) | Developed the theory of "zyzytiology". Studies of the importance of electrolytes in heart disease. |  |
| **René Leriche (FR)** | 1930-1953 | 79 | J. Roux-Berger (FR) et al. | Work on the physiological and therapeutical effect of resection of obliterated arteries. | 1936  1943 |
| **Léon Frédéricq (BE)** | 1930 | 2 | Albert Lemaire (BE), F. D. Hollander | Work on the physiology of the heart and respiration, ecpecially considering hemocyanine. |  |
| **Jean François Heymans (BE)** | 1931 | 5 | O. Rubbrecht (BE), Alexandre Besredka (BE), F. de Beule (BE), Paul van Durme (BE), N. Goormatigh (BE) | Work on the physiology and pharmacology of respiration and blood circulation |  |
| **Heinrich Hering (DE)** | 1932-1937 | 6 | A. Güttich (DE), Paul Hoffman (DE), Geza Mansfield (HU), A. Durig (AT), A. Policard (FR), J. Collet (FR) | Work on the function of the control of blood pressure, Work on sinus caroticus. | 1934 |
| **Hermann Rein (DE)** | 1933-1951 | 9 | Ludwig Aschoff (DE), C. Noeggerath (DE), W. Nonnenbruch (CZ), Martin Ficker (BR), H. Schottmüller (DE), K. Bürker (DE), Carl Hiller (US), Ludolph Brauer (DE) | Work on blood distribution in higher organisms. |  |
| **Corneille Heymans (BE)** | 1934-1939 | 8 | A. Durig (AT), P. J. Hanzlik (US), B. Krishnan (IN), Geza Mansfeld (HU), Alexandre Besredka (BE), J. Bouckaert (BE), A. Vandevelde (BE), Frans Daels (BE) | Work on the regulation of respiration and blood circulation; Work on the influence of sinus caroticus on the rate of the heart beat, and on blood pressure and respiration. | 1934 1936  1939 |
| **Nikolaus Anitschkov (RU)** | 1937 | 1 | Pierre Nolf (BE) | Work on the experimental production of arteriosclerosis |  |
| **Harry Goldblatt (US)** | 1938-1950 | 16 | P. Schwartz (TR), C. Oehme et al. | Studies on permanent hypertension; The connection between renal ischemia and hypertension | 1941 |
| **Makoto Ishihara (JP)** | 1939 | 1 | Daize Ogata (JP) | Work on "Heart-Action". |  |
| **Efim Semenovich London (RU)** | 1939 | 2 | A. Voynar (RU), A. Calisov (RU) | Proposed the methods of angiostomy and organostomy. |  |
| **Irvine Page (US)** | 1941-1942 | 3 | L. da Cunha Motta (BR), Walter Meek (US), Arthur Tatum (US) | Studies on experimental arterial hypertension; Isolation of renin and angiotonin. | 1941 |
| **Arthur Stoll (CH)** | 1941-1953 | 4 | F. Sternon (BE), F. Schoofs (BE), Paul Hermann Müller (CH) | Chemical composition of heart glycosides, especially scillarens, digitalins and strophanthidins. | 1941 |
| **Alfred Blalock (US)** | 1947-1953 | 29 | R. K. Ghormley et. al. | The surgical treatment of malformations of the heart. | 1947 1949 |
| **Helen B. Taussig (US)** | 1947-1953 | 24 | R. K. Ghormley et. al. | The surgical treatment of malformations of the heart. | 1947  1949 |
| **Norbert Goormaghtigh (BE)** | 1947 | 1 | F. Derom (BE) | Significance of afribil muscle cells for arterial hypertension | 1947 |
| **Clarence Crafoord (SE)** | 1948-1953 | 5 | Afik Chakar (TR), Niels Dungal (IS), Helge Wulff (SE), W. v. Brunn (DE), L. Christophe (BE) | Surgical treatment of congenital cardio-vascular abnormalities; Surgical treatment of isthmus aortae |  |
| **Robert Gross (US)** | 1949-1952 | 5 | George Whipple (US), Niels Dungal (IS), Helge Wulff (SE) | Experimental studies, and surgical treatment of malformations of the heart; Surgical treatment of isthmus aortae | 1949 |
| **Claude Beck (US)** | 1949-1952 | 3 | George Whipple (US) | Experimental studies, and surgical treatment of malformations of the heart. | 1949 |
| **Frank Wilson (US)** | 1950-1952 | 2 | P. Formijne (NL), Guillermo Allende (?) | Fundamental theoretical and practical investigations on the electric phenomena of the heart |  |
| **Gustav Nylin (SE)** | 1951 | 1 | Niels Dungal (IS) | Surgical treatment of isthmus aortae |  |
| **Jacob Jongbloed (NL)** | 1951 | 4 | E. Janssen (ZA), G. Morin (FR), G. Jayle (FR), Heckenroth (FR) | A mechanical device for heart function during cardiac surgery of malformations |  |
| **Werner Forssmann (DE)** | 1952-1953 | 3 | N. Guleke (DE), William Murphy (US), Ludwig Heilmeyer (DE) | No Motivation (Annotation: 1956 Nobel Prize Laureate “for (their)discoveries concerning heart catheterization and pathological changes in the circulatory system.”) |  |
| **Carl Wiggers (US)** | 1953 | 1 | Corneille Heymans (BE) | No Motivation (Annotation: Cardiovascular physiologist) |  |
| **André Cournand (US)** | 1953 | 1 | William Murphy (US) | No Motivation (Annotation: 1956 Nobel Prize Laureate “for (their)discoveries concerning heart catheterization and pathological changes in the circulatory system.”) |  |

Table 2 (supplement): Longlist of cardiovascular nominees 1901-1953
